# Exploring the typology of decision-makers, institutions, and incentives that shape health decisions in Pakistan and insulate decision makers from citizens feedback

**DOI:** 10.3389/fpubh.2023.1253798

**Published:** 2023-12-08

**Authors:** Adnan A. Khan, Romesa Khan, Zainab Khawaja, Muhammad Ibrahim, Zarnab Shaheen, Ayesha Khan

**Affiliations:** ^1^Research and Development Solutions, Islamabad, Pakistan; ^2^Ministry of National Health Services, Regulation and Coordination, Islamabad, Pakistan; ^3^Akhter Hameed Khan Foundation, Islamabad, Pakistan

**Keywords:** evidence-based policymaking, decision-making, political economy, policy ambiguity, Pakistan government, implementation science, public choice

## Abstract

**Introduction:**

In developing countries such as Pakistan, program and policies underperform in providing public good as weak institutions lead to decisions that are unresponsive to citizens and are driven by personal motivations of the incumbents. We describe the decision-making processes in the health sector through the framework of “Public Choice” theory which posits how individual motives shape institutional performance and direction.

**Methods:**

We conducted 84 qualitative interviews with five types of stakeholders: politicians, senior and mid-level bureaucrats, donors, public health experts and media personnel spanning 2 periods over a decade. The initial interviews were conducted during 2013–2015 period and a case study on decision-making during the COVID-19 response was added in 2020–2022 period.

**Findings:**

Most new ideas originate from top political leadership, guided by personal agendas or political expediency. Senior bureaucrats implement politicians’ agenda and mid-level officials maintain the status quo and follow established “authority.” Since officials’ performance, promotions, transfers, and the rare dismissals are based on tenure deviations rather than work performance, individuals and institutions are reluctant to take initiative without “consensus” among their colleagues often leading to inaction or delays that obviate initiative and reform. The public sector lacks institutional memory, formal information gathering, and citizen engagement, impacting public goods, health services, and policies. Media and donor personnel are important influencers. However, media mostly report only “hot issues” in health, with short publication and reader attention cycles. Donor personnel are the most likely to follow evidence for decision making, albeit while following their institutional priorities determined centrally. The COVID-19 response is presented as a contrast from usual practices, where evidence was used to guide decisions, as the pandemic was perceived as a national threat by the highest leadership.

**Conclusion:**

Absence of citizen feedback and formal systems for evidence gathering and processing leads to decisions that neglect the needs of those they serve, prioritizing personal or political gains and perpetuating the status quo. However, the COVID-19 pandemic emphasized the importance of evidence-based decision-making and offers valuable lessons for reforming decision-making processes.

## Introduction

In a world of scarcity, all decisions would ideally be based on empirical evidence. However, this can be challenging, particularly in developing countries, where many health and social problems compete for scarce resources, while governance, competencies and other constraints limit the effectiveness of programs and policies (1–3).

Pakistan is unusual for a low-middle-income country in that it underperforms in health and development outcomes given its level of human capacity and nominal political will. Local experts attribute this underperformance to factors such as a lack of funding, infrastructure (health systems), poverty, population size and decentralization stemming from devolution of health services to provinces as part of the 18^th^ amendment to the constitution of Pakistan (4). However, empirical evidence does not necessarily support these observations (5–7). Other commentators have blamed political interference (8) for policies that ignore problems of the larger public (9). On the other hand, Pakistan managed its COVID-19 epidemic better than most countries, suggesting that it can take effective decisions in health and beyond when it must (Khan et al., under review).

Evidence use is a three-step process that includes production of evidence, understanding of the evidence by decision-makers and conversion of that understanding into policy that should ideally improve the life of citizens. Much debate on evidence use in policy draws on lessons from evidence-based medicine (10), however there are clear differences (11, 12). For one, EBM is directed toward doctors or other medical practitioners who have been trained in science, while most policy makers aren’t scientists. Also, the level of proofs in medicine are more precise than in social or policy space. For example, is Drug (or procedure) A superior to Drug B based on a randomized control trial (RCT). In public policy, most evidence must be inferred from diverse sources many of which may be imprecise, while nuancing it for culture and context. This is further complicated by the fact that politicians (and bureaucrats), who are not necessarily trained in evidence interpretation, manage, and must make concessions to competing interests. Not surprisingly then, empirical work shows no difference in evidence uptake by education or expertise of the policymaker, type of policy to be made and the means by which it is produced (12). Similarly, many attempts at increasing the capacity of decision-makers to understand or take up evidence, or measures that enhance the dissemination of evidence to policy makers, have not led to better utilization of evidence in policy or translation into effective programs (12, 13).

We used a “Public Choice” framework to understand decisions in the health sector. Public Choice uses an economic lens to understand decisions. In this view, individuals are considered as boundedly rational (i.e., logically consistent) actors that seek to maximize their own benefits, and that the decisions by an institution reflect the sum of choices made by people who work for that institution (14, 15). Thus, when there is robust citizen feedback, decision-makers respond by promoting public goods to remain in power. However, when such feedback is weak, decision-makers follow private gains or vested interests that may be indifferent or even contrary to the interest of citizens that they must be serving (15).

We explored the processes of how decisions in health are initiated and implemented and what incentives motivate them in the context of types, and hierarchy of political decision-makers. We use the health sector in Pakistan as an exemplar of decision making in a low-middle income country using the Public Choice framework. Decision making is described for both when the sector underperforms in its routine work and when it succeeded, as in case of Pakistan’s COVID-19 pandemic response.

The initial interviews were conducted until 2015 as part of a grant to promote evidence to the public sector and other decision makers. These findings showed the state of “normal” decision-making under routine circumstances. Since decision-making during the national response to COVID-19 appeared very different, both since the outcomes of decisions became apparent quickly and because there was a direct public feedback on government policy, interviews of decision makers from this period were added as a case study of a contrasting paradigm. During these interviews, government colleagues and their public health interlocutors felt that once the COVID-19 response subsided, and for routine work even while COVID-19 pandemic was ongoing, decision making appeared to have reverted to the routine pre-COVID-19 paradigm, therefore, no further interviews were conducted for the post COVID-19 period.

## Methodology

RADS has engaged with the public sector to advocate using evidence for decision-making since 2011. This engagement informed the creation of categories of respondents we wanted to interview. Due to the nature of their work and stature, convenience sampling had to be employed; the selection of actual individuals was based on name recognition and through introductions.

Two categories of decision-makers were interviewed: Politicians interested in or having worked with social sector issues particularly health, senior government officials (bureaucrats: grades 21 and 22), and mid-level officials (grades 18–20) working in health. Three types of influencers were interviewed. Media personnel interested in health issues, academics that sometimes advise the government, and local staff of donor agencies working in health at the time (Table 1).

**Table 1:** Sample disaggregation.

	Federal	Sindh	Punjab	Total
Politicians	4	3	7	14
Govt. officials—senior	5	4	8	17
Govt. officials—middle	4	3	5	12
Donor officials	8	2	2	12
NGOs	4	3	4	11
Professional bodies	2	2	2	6
Media	2	3	3	8
Academia	1	2	1	4
Total	30	25	30	84

The study tool was a semi-structured instrument, that was adapted from a previous evidence to policy initiative. The formal study included 84 interviews (Table 2) from Sindh, Punjab, and the Federal capital (Table 2) during 2013–2014. The inquiry was around four key questions: (1) how and which decisions are made in health and population and at which level of organizational hierarchy do they originate, (2) how are needs for information recognized, (3) once recognized, what are the main sources of information, and how is the information processed, and (4) how information is used (or not) for decision-making. Due to the sensitive nature of the respondents’ political positions and a general reluctance among government officials to allow recordings, no audio recordings were made. Instead, the interviewers took notes during the actual interviews that were later expanded. The data from these transcripts were organized by respondent type (decision-maker and influencer) and was analyzed in light of relevant themes for each respective respondent type. Approval for the original study protocol was provided by the National Bio-Ethics Commission.

**Table 2:** Themes for interviews.

Themes for decision-makers	Themes for influencers
What are the information sources? How is information prioritized/validated? Limitations of this process? Who do they trust? Who/what (does not) influences them? Who actually makes the decisions? What are their motivations/incentives? What are they doing? And who’s doing it? Limitations? What are they not doing? Why? Limitations?	What is their role in influencing decision-makers? What are their motivations? Where do they obtain their information? How do they prioritize/validate information? What are the limitations of this? What is their role in beneficiaries’ feedback loop?

The second part of the study included Key Informant Interviews (KIIs) with four individuals, including high level personnel from Ministry of Health (MoNHSRC), the Ministry of Planning and Development and military personnel deputed to the National Command and Operation Center (NCOC) for the COVID-19 response, who had participated in decision-making at the NCOC. A semi-structured tool was developed for this round of interviews, based on the themes from the first round, but questions were tailored to focus on decision-making during the COVID-19 pandemic.

Information from interviews was supplemented with observations by lead author (AAK) who had supported and attended nearly all sessions of the NCOC. These interviews were conducted in the summer of 2022 and observations for the case study to cover NCOC decision making from October 2020 to June 2022. This addition was approved by RADS internal IRB.

## Results

### What kind of decisions are made and by whom?

Decisions and decision-makers fall into two distinct categories: political leaders and (senior and mid-level) government officials. Most policy level, paradigm changing or “heresthetical” (16) decisions are taken by senior politicians such as party leaders or the inner core around them. The inner core is instrumental in providing “seed ideas” for new programs according to their party’s political agendas or political expediency. These are usually high visibility “headline projects,” i.e., major programs, construction of hospitals/clinics or by providing private goods such as individual jobs to potential voters. Major examples include two large social protection programs, the Lady Health Workers Program and the Benazir Income Support Program that were both initiated on direct orders from the then Prime Minister. Similarly, in Sindh, the health department was separated between a primary care and a curative care section at the direction of the Chief Minister. Junior politicians, including members of national or provincial assemblies or senators, often raise queries on health programs but generally do not have the political capital or persistency to ask for such changes, or follow results, and hence defer central direction to party leadership.

Senior officials face three challenges. Firstly, managing transfers and personnel issues consume most of their time, leaving little time for immersion in the health program or providing innovations. Secondly, their own frequent transfers lead to short termism in both the administrative wing led by the Secretary and the technical wing headed by the Director General (DG) for slightly different reasons. Secretaries tend to transfer to another Ministry within a year, with an average tenure of 5 months for the Secretaries at the Population Welfare Department in Punjab in the 3 years prior to the study. The Director General is a ministry employee. While they cannot transfer out to another ministry, they can still be transferred between the federal and provincial governments. For either, there is often insufficient time to familiarize themselves with nuances of their various posts and to stay for long enough to implement the changes they conceive or to see some results. The third issue is that of understaffing, that unduly affects the technical wings of ministries. Due to under-recruitment, transfers, and leaves, at any given time, only around half or fewer technical officer positions are occupied. For example, at the time of writing, only 6 of the 21 sanctioned officer positions were filled at the federal ministry. This means that seniors are overburdened with routine work—which is often expedient—and putting out fires, leaving little time to reflect, peruse evidence or innovate.

Mid-level government officials stay in their departments for years and form institutional memory. They mostly make minor implementation decisions, such as temporary personnel assignment at facilities, and seldom participate in discussions of innovative programs or policy. The fear of repercussions such as transfers to unfavorable locations or jobs, if outcomes turn bad, prevents them from voicing different opinions to the proposed programs. For example, some officials complained about repeated cycles of the same program by different donors, but none voiced their critique to their bosses. Respondents felt that acts of commission are more likely to be punished as compared to acts of omission, leading them to choose inaction or silence over innovative action even when they disagreed with the suggested programs or policies. Many mid-level government officials felt that policies are dictated by top bureaucrats, suggesting that the middle management of ministries does not directly interact with the political leadership, as access to the top leadership is very hierarchical. Politicians usually communicate (i.e., non-policy) decisions verbally, while government officials follow protocols, i.e., paper files. Documentation protects officials to some extent from the repercussions of illegal orders and allows for accountability but can also be used to induce delays and inefficiencies.

### Factors that affect decision-making

We found that five major factors affect higher-level decision-making: resource availability, job insecurity, personality of the administrator, direct relationship with the decision-maker, and donor priorities. Nearly all government respondents felt that limited resources restrict hiring of adequate “technical” staff in their offices, and they must rely on donors and NGOs for personnel, technical expertise, and supplies. This opens government planning to influence from shifting donor priorities rather than establishing clear longer term institutional “roadmaps.”

Senior officials, who have short tenure, feel that job insecurity forces civil servants to align their interests with those of the political parties and politicians. Most government employees spend time in “job saving” by building relationships, and not offending people even at the cost of program effectiveness.

*“Tenure is absent and this really influences decision making. If I am not sure when and why I will be dismissed, I spend most of the time in “job saving” keeping people happy.”* (Government official).

*“At the policymaking level, job insecurity forced the civil servants to align with the political parties. Successive governments brought in their favorite civil servants to occupy key positions. Loyalty rather than competence became the acid test for survival. The winners in this game included political leaders and acquiescent civil servants while the losers were ordinary citizens who ceased to have access to the government and had no way of having their grievances redressed.”* (Government official).

This is an indirect reflection of promotions and job security being tied to tenure, arbitrary or non-merit-based evaluations that do not consider programmatic performance or citizen feedback of programs and policies, or even power dynamics between senior officials and politicians. In the absence of institutional frameworks for decision-making, “personality of the administrator” drives decisions. In fact, our respondents cited multiple occasions of programs guided by personal interests of the official in charge, without formal assessments of need.

*“A women’s health program funded by Asian Development Bank was initiated and guided by personal interests but there was no concrete documentation of the need for it on the ground. This program later on became the foundation of MNCH program.” (Government official).*

*“When there is no framework the personality of the administrator is the driver, the leading influence (or lack thereof) in making decisions. Health [sector] has suffered, due to lack of (institutionalized) decision-making” (Government official).*

### No formal evidence management system

Nearly all decision-makers endorsed the importance of using research evidence in policy formation, but few could cite examples of such use in their own decisions. Yet others felt that data are “useless” for policy makers, or that research or evidence are theory and therefore impractical for their real-life decisions. Upon probing, we realized that they do not have the time, skills, or supportive systems to process this information, and they ignore or discredit what they do not understand. Sometimes this is addressed by delegating to expert committees. In effect, with little emphasis on “reading” and subject knowledge, decision-makers form their opinions based on personnel experiences and (sometimes insufficient) knowledge. Once committed, they then seek out information and information providers that support these ideas, perpetuating confirmation bias.

*“Many matters are not shared. Information that comes in is disjointed and in pieces so that we do not always know what is going on. And often we are too busy (with day-to-day issues) to pursue each lead. If there was a system of information sharing … it would be easier” (Politician).*

A decade ago, there were no formal evidence collating mechanisms at either the federal or provincial levels. Since then, some versions of health systems or policy units have been deployed by the federal and provincial governments, however, they are all in early stages of development. Mostly, they track data, sometimes in simple dashboards etc., to give the minister or other senior officials a quarterly or semiannual report. Nevertheless, we seldom found higher functions such as data quality checks, regular use of data to follow progress, identifying program issues, making estimates or projections, or using data to make a case for more funding and to report about the progress of funded projects; and none of the information processing for decisions is done routinely.

*“There should be a platform for exchanges and information sharing, or some institutional mechanisms where data can be fed in. Like a central repository of knowledge!” (NGO personnel).*

All of this translates into an absence of formal systems to process and translate evidence into decisions. Information received is inaccurate or fragmented and is either not used or remains under-analyzed. More importantly, there are no mechanisms to garner citizen (end-user) feedback into the decision-making process, resulting in policies that are indifferent to the rights and needs of citizens. No government official in our study could recall ever having discussed the impact of their policies or decisions with community beneficiaries of their decisions. Thus, irrespective of causes, policy formulation is often arbitrary, *ad hoc*, inconsistent, and indifferent to citizen’s needs, particularly the poorest populations.

*“Corrective actions or feedback to the problems is rare. The Secretary is accessible only to top management and often does not see or hear what the real issues are. Meanwhile, each subsequent chain makes the story better, so that by the time it reaches the top everything is going wel—always.” (Government official).*

### The role of influencers

Direct contacts or relationships play a significant role in decision-making in Pakistan. Decision makers repeatedly reported consulting personal acquaintances, such as friends or family with a shared past, that were trusted as well-wishers, but do not necessarily have relevant expertise in the field. Confidentiality, reliability, and duration of the relationship predicted the likelihood of trust in the advice received. The advice then given is viewed in the skeptical light of political expediency and visibility for the individual. This can work for or against good decisions. For example, one respondent said,

*“Breast cancer awareness has now become a part of routine care in many government hospitals. This notification was pushed through due to personal relationship of a government official with a cancer patient in the immediate family.” (Government official).*

Paucity of research funding is a major constraint evidence generation. Another is that the public sector higher education system favors time spent on teaching and allows none for research. Furthermore, there are no rigorous, objective, or formal mechanisms of scoring reliability of information produced. Not surprisingly, we found no examples of research-based input being utilized in decision making or to amend adverse policies or programs.

Communication between academics and policy makers is limited, although renowned academics from nationally recognized first tier universities participate in a somewhat tokenistic dialogue. Most are senior doctors with an interest in health systems, and few have experience of research or evidence use. There are few researchers interested in evidence translation from data, and they seldom interact with decision-makers. For their part, many academics do not see their actual role in interpreting research for policy makers beyond participating in numerous, *ad hoc* “consultation meetings” often arranged by donor-supported programs. However, few busy decision-makers (politicians or senior officials) stay long enough to hear the presentations or discussions. Academics felt that politicians do not share their political priorities with them and do not engage in a dialogue, seeking only to receive suggestions that confirm previously held positions. They also find top bureaucrats to be inaccessible or minimally attentive. Some civil society stakeholders engage with decision-makers, although others expressed suspicions that limited engagement with select information brokers may be intentional to “avoid sharing power” and those that are included form a version of “incumbent capture” creating “echo chambers.”

*“(Policymakers have) deep mistrust of academics and research, (and because of) supposed irrelevance between theoretical domains and practical knowledge, they actually take pride for having practical knowledge and can do without the theory base.” (Academic).*

Donor personnel (local and international counterparts) are often the most influential in government decisions, by providing well-articulated ideas that they sometimes support financially. In most cases, local staff of donor agencies are well read, receive clear guidance about their institutional priorities and are supported by specialist personnel at their home country offices. Government officials mentioned that donors have their own predetermined and sometimes shifting agenda, however they feel that it works in everyone’s “mutual interest” to work together since it leads to novel programs, refurbished offices, and equipment etc. (institutional benefits) but also trips, and conference visits (personal gains). Some officials reported that donor suggested programs from other country context that may not be applicable in Pakistan, but in many such cases, officials opted to remain silent despite having concerns about utility or efficacy of such programs.

Media influencers create two-way information sharing platforms to report news and allow citizens to voice their opinions regarding policies. Journalists may be “beat” reporters who are often junior and report on daily events. More senior ones are often columnists or TV anchors that write/report on issues. For reporters, health is only important when there is a newsworthy event such as an outbreak, epidemic etc. A “hot event” often leads to a press conference, which is covered in print and social media, and receives heavy coverage if the minister attends it. Senior reporters acknowledge that health is a major area of interest for the public but feel that often there is insufficient material to write from, as information from ministries is inadequate. Ministries have at most 1–2 people with a part-time responsibility to work with the media. Journalists that publish positive stories are favored and are invited repeatedly, further consolidating echo chambers around decision-makers.

Journalists feel that in-depth and series reporting on the same topic is preferred by only a niche audience. Most consumers prefer headline sensations and bore quickly with depth of reporting, which shortens the “attention span” on health issues contribute to a highly competitive commercial environment. While journalists said that they verify all information by sampling the public opinion, their sampling is done by convenience with its inherent limitations driven by time, finances and whom they know.

*“Media role is limited by depth of knowledge and formal education on responsive journalism, weak rewards for pursuing depth, funding from sources that are connected to government and other vested interests; and independence of reporting is questioned.” (Journalist/Columnist).*

*“Stories can be withdrawn if the external pressure is strong—editors will “scold” and in extreme cases marginalize reporters who portray an unfavorable picture particularly of government efforts.” (Reporter).*

*“Have to be careful and sensitive to the reputation of the paper and audience (particularly political leadership and the social environment of that time) catered to.” (Reporter).*

### Interactions between ministries

Our respondents stated “asking questions in the national or provincial assemblies” is a key medium for politicians to interact with ministries. These queries relate to program numbers, impacts, or pure logistical, staffing, and hot issues such as adverse events in hospitals or with epidemics. Since the timing and format of such queries is often *ad hoc*, it requires that ministry officials stop their routine work to respond. The *ad hoc* format also adds confusion for what and how to answer, as some questions are beyond the scope of information available. Ministries such as Health, Population Welfare, Finance, Commerce and Industries, and Planning, seldom interact or collaborate. One senior decision-maker explained that there is no reason for ministries to collaborate, as their work, budgets, policies, and programs are independent. Even when there are the occasional formal agreements to cooperate, information and activities usually remain siloed.

Ruling political parties seldom engage opposition politicians in deliberations to avoid conflicting viewpoints and sharing of “political credit.” This is mostly done by denying the latter access to government data and maintaining secrecy of programs and policies until final deployment. For example, one of our respondents called the budget “*the number one secret document*” even though it is within the ambit of the opposition to debate and question it on the floor of the house. Another respondent said,

*“We make our inferences, particularly from what data are withheld by the government, and then work backwards to identify what is being hidden.” (Politician).*

### Interactions with citizens: does a feedback loop exist?

Communication between government officials and the end-users or citizens they seek to serve is absent. Our respondents reported no medium to solicit feedback from the public and none could cite even one instance when public opinion and feedback changed the practices of top or mid-level decision-makers. Some government officials felt that such a feedback loop would only be “superficial and unnecessary” since our population is uneducated and therefore unable to make reasonable personal choices. So, while several politicians from both major parties, have raised awareness and mobilized communities for girl child education, delaying age of marriage, limiting family size and maternal health, they too feel that in public policy, the information only flow in one direction, from them to people, since “communities do not really know what is best for them” and therefore, must be told what to accept. A culture of denial also creates disconnects. As one influencer stated, “*providing feedback to government authorities on their policies, malpractices, and performance is akin to ‘catching the bull by the horns’ and can lead to being marginalized by decision-makers.”* This “yes sir” culture creates echo chambers where only filtered information reaches the top and real issues are brushed under the carpet.

### COVID-19 in Pakistan: a case study in contrast

Once the COVID-19 pandemic hit Pakistan in early 2020, a National Command and Operation Centre (NCOC) was established to effectively manage the pandemic. It was co-led by the minister of planning, a senior military general and the health minister. Other ministries such as education, industries etc. were invited regularly as needed. The NCOC was a largely a federal body but met regularly with provincial authorities and made decisions to manage the epidemic in the entire country. While the NCOC was empowered to make rapid decisions, the deliberations were democratic and all ministries, provinces, media, and the civil society, including trade or industry groups etc., were invited to discussions to ensure ownership. Implementation and follow up of decisions were by military personnel specifically assigned to the NCOC. Data from the field were collected by provincial health departments feeding into the NCOC datasets. Ministry of health and military teams analyzed the data to produce inferences and recommendations for NCOC leadership. Recommendations were debated in depth and participation was encouraged at the junior analyst level. The authors of this paper participated in the daily NCOC team meetings and closely observed the processes.

*“From what I know, previously decisions were based on political interests, but later during the COVID response, the opportunity cost of every decision was discussed. For example, on the one hand, data is telling us that if we close schools then COVID cases will drop, and we can save lives. But students need their education too and they’ll have to stay at home for another year. Even their parents were tired of them staying home all day. All aspects were discussed.”* (Ministry personnel).

NCOC experience contrasts sharply with much of our previous findings. The response was led by evidence, which was sought systematically, continuously and with a degree of skepticism for “too good to be true” approach. Decisions were inclusive and extensively debated. On many occasions, the leadership changed their mind when presented with contrary evidence by provincial authorities, personnel from different ministries or even junior analysts or implementation teams. Even in cases of dissent from provinces and ministries, all parties agreed to allow the NCOC to be the final arbiter. Our respondents felt that this was because ministries and provinces understood that the NCOC had done due diligence and because COVID-19 had raised the stakes, putting everyone’s “skin in the game.” In the same vein, once the epidemic abated and the NCOC’s functions were transferred to the (National Institute of Health) NIH within the MoNHSRC, information sharing became less transparent, and engagement with provinces and ministries eventually receded to pre-COVID-19 levels.

*“We used to discuss these things… what our evidence and information shows, as well as what the public’s sentiments are or what the public says. That’s how I think it was different, because previously, and even afterwards there were no discussions or debates or anything like that.”* (Ministry personnel)

## Discussion

We describe decision-making in health using the “Public Choice” lens, which explains how actions of rational actors add up to the outcomes within a political system (15). The most striking finding is the universal absence of feedback from citizens or beneficiaries of programs. This leads to lack of consideration of program outcomes, either no or piecemeal measurement of program performance (results), and no one has a clear picture of the situation or the outcome of programs. Absent a need to produce results, decisions become individual driven and *ad hoc*, particularly as they occur in a context of transient and arbitrary appointments, permanency of government tenure with incentives to avoid mistakes of commission, but not of omission, and the lack of formal measurement of performance. The lack of a focus on outcomes allows incumbent capture, avoidance of innovation or technical outsiders and limited interactions with evidence brokers such as academia. Decision-making is tiered. Innovations or reforms originate from top political leadership, while senior and mid-level government officials implement them, but seldom innovate.

Although the use of evidence in policy in health is sometimes considered synonymous with evidence-based medicine, the latter uses precise scientific data and its practitioners are science trained professionals such as doctors or nurses, while policy making involves complex interaction of politics, social culture, institutional arrangements and processes, incentives, or values of diverse groups of people, and is the purview of politicians or bureaucrats with generalist backgrounds, that aren’t necessarily trained in science and have varied agendas (2, 10–12, 17).

In our study, the lack of trust of available data among our respondents may be an implicit acknowledgement of the poor quality of data available, the inability of frequently transitioning officials to understand domain-specific data and the lack of institutions that can synthesize data into actionable information. Trust is the belief in the reliability of something (or someone) and facilitates early-stage social order allowing people to cooperate for work (18). Scientists may be seen as unbiased and as expert, particularly when they can provide succinct advice that minimizes ambiguity (12), and collaboration between researchers and policymakers can be the best facilitator of eventual evidence use (13, 19). Our respondents often found academics too abstract and despite increasing amounts of evidence produced, as seen from an increase in published journal articles in PubMed from Pakistan, from 1,416 in 2010 to 15,008 in 2022, hardly any policy maker reported being approached by academics or vice versa in a meaningful manner. Decision-makers adapt to these limitations by turning to personal, sometimes unscientific, sources that they trust socially in a divisive political environment, or to committees that can garner specialist help but also dilute (consensus) responsibility. In essence they act like rational actors that hedge their bets (18).

We found that the types of decisions vary by hierarchy. Politicians bring innovations driven by their party agendas, private goods and less by citizen needs or public goods. Given short tenures, senior officials seldom make major changes or invest themselves in ensuring best outcomes for citizens, that require long-term planning and execution. Over time this short-term thinking has become the norm at ministries and changing the status quo is anathematic. This has significant implications for information brokers and system reforms. Efforts to conduct short term training or establish Policy and Data Units without understanding the context are unlikely (as has been seen) to promote evidence use by decision-makers. To do this, evidence brokers must revisit how the incentives of decision makers and citizens can be better aligned, then try and meet the information needs of decision-makers in this context including rigorously measuring if information was actually used (20, 21).

Throughout this study, citizen feedback loops were absent, and not missed by officials. While Politicians are more attuned to moods of their constituents, this is at an instinctive level and does not connect with formal systems to channel this feedback at the institutional levels. “Policy feedback loops” refers to a channel through which some of the results and consequences of a certain policy are incorporated into the framing of subsequent policies in a cyclical process (22). Leutert describes the use of grassroots feedback to shape China’s experiment to shape their economic reforms in the 1970s and 80s, in what is largely a very top-down governance structure. China’s example is especially pertinent since decision-making in many developing countries including Pakistan is top-down, and because it exemplifies how ground up citizen feedback was used in China.

This suggests another important principle, “skin in the game.” Essentially people are invested in situations when they have something to gain or lose. Absence of public end users from feedback loops also exemplifies the lack of impact of opinion of the public on policy. This is consistent with the basic framework of Public Choice Theory that governments will only listen to their people when the latter are empowered (14, 23), especially economically empowered (24) or living in a functioning democracy. Given that only around 13% of the world’s population lives in a functioning democracy, and another 33% under a flawed one (25), the absence of citizens in feedback loops on decisions is perhaps more common than is appreciated. Under these circumstances, decision-makers only respond to those that can directly benefit or harm them. Not surprisingly then, job security and political patronage were prioritized over results, value for money and given the arbitrary influence of personal influencers, the decisions are arbitrary and not very evidence based.

All this was turned on its head in Pakistan’s response to the COVID-19 epidemic when “skin in the game” was in controlling infections and deaths in the face of competing priorities such as keeping the economy running and schools open, when Pakistan was already been struggling economically and food riots/civil strife was a likely possibility (26–28). Ineffective management of the pandemic would likely have pushed the country beyond a precipice that top leadership felt they could not afford to cross. An elaborate set of new and older structures were vitalized to control the pandemic, and this was done effectively, as seen by some of the fewest infections and deaths globally (29). However, once the danger abated, lessons in evidence-based decision-making were not incorporated in the country’s health sector which then reverted to its pre-COVID-19 norms.

## Limitations

This study was originally conceptualized in 2013–14 as a part of an evidence to policy initiative to better understand how the evidence produced was being utilized. It was further supplemented over the last 3 years using the same conceptual framework and tools. It is possible that the context and therefore its implications on decision making may have changed between the original interviews and the Covid case study. This is a qualitative study based on interviews of individuals that were approached and/or accepted discussions. It is possible, but perhaps less likely, that we may have systematically missed important concepts or themes from individuals that did not participate, or we could not access.

## Conclusion

Our study shows individual driven, arbitrary, and *ad hoc* decision-making in Pakistani health system, it also suggests avenues for improvement when circumstances dictated the need. So, while an abundance of evidence is produced, it is seldom translated for or used by decision-makers. This suggests a critical revisiting of the information use and incentives paradigms to create a culture where information use matters and citizen feedback is a foundational component for government officials beyond just tokenism (30). Some of this may come from better interpretation of national and provincial data that are already available but can be enhanced by collecting client feedback at services, to involve communities in local governance of services, working with journalists to understand and report on complex issues for the public and perhaps devising pilots of academic researchers collaborating with social media influencers to customize a language that can resonates with the public. All of this must be done in the political economic context of evidence use and its constraints, and by implicitly understanding that embedded knowledge brokers in ministries have longer tenure than other government officials and can mediate and sustain some of these reforms through perseverance and better alignment of bureaucratic incentives.

## Data availability statement

The raw data supporting the conclusions of this article will be made available by the authors, without undue reservation.

## Ethics statement

The studies involving humans were approved by National Bio-Ethics Commission, and RADS IRB. The studies were conducted in accordance with the local legislation and institutional requirements. The ethics committee/institutional review board waived the requirement of written informed consent for participation from the participants or the participants’ legal guardians/next of kin because it was acknowledged that government employees do not sign forms.

## Author contributions

AAK: Conceptualization, Formal analysis, Funding acquisition, Methodology, Supervision, Validation, Writing – review & editing. RK: Data curation, Formal analysis, Validation, Writing – original draft, Writing – review & editing. ZK: Data curation, Formal analysis, Validation, Writing – original draft, Writing – review & editing. MI: Project administration, Supervision, Writing – review & editing. ZS: Data curation, Validation, Writing – original draft. AK: Conceptualization, Funding acquisition, Methodology, Project administration, Supervision, Writing – review & editing.
